# Expression of alternative developmental pathways in the cabbage butterfly, *Pieris melete* and their differences in life history traits

**DOI:** 10.1002/ece3.5731

**Published:** 2019-10-21

**Authors:** Jian‐Jun Tang, Hai‐Min He, Shao‐Hui Wu, Cao Zou, Fang‐Sen Xue, Lan Xiao

**Affiliations:** ^1^ College of Computer and Information Engineering Jiangxi Agricultural University Nanchang China; ^2^ Institute of Entomology Jiangxi Agricultural University Nanchang China; ^3^ Department of Entomology University of Georgia Tifton GA USA; ^4^ School of Education Huazhong University of Science and Technology Wuhan China; ^5^ Foreign Language School Jiangxi Agricultural University Nanchang China

**Keywords:** bet‐hedging, diapause, direct development, life history trait, *Pieris melete*, temperature

## Abstract

The seasonal life cycle of the cabbage butterfly, *Pieris melete* is complicated because there are three options for pupal development: summer diapause, winter diapause, and nondiapause. In the present study, we tested the influence of temperature, day length, and seasonality on the expression of alternative developmental pathways and compared the differences in life history traits between diapausing and directly developing individuals under laboratory and field conditions. The expression of developmental pathway strongly depended on temperature, day length, and seasonality. Low temperatures induced almost all individuals to enter diapause regardless of day length; relatively high temperatures combined with intermediate and longer day lengths resulted in most individuals developing without diapause in the laboratory. The field data revealed that the degree of phenotypic plasticity in relation to developmental pathway was much higher in autumn than in spring. Directly developing individuals showed shorter development times and higher growth rates than did diapausing individuals. The pupal and adult weights for both diapausing and directly developing individuals gradually decreased as rearing temperature increased, with the diapausing individuals being slightly heavier than the directly developing individuals at each temperature. Female body weight was slightly lower than male body weight. The proportional weight losses from pupa to adult were almost the same in diapausing individuals and in directly developing individuals, suggesting that diapause did not affect weight loss at metamorphosis. Our results highlight the importance of the expression of alternative developmental pathways, which not only synchronizes this butterfly's development and reproduction with the growth seasons of the host plants but also exhibits the bet‐hedging tactic against unpredictable risks due to a dynamic environment.

## Graphical abstract

The expression of developmental pathways in the cabbage butterfly, *Pieris melete* highly depended on temperature, day length, and seasonality and exhibited a bet‐hedging tactic. Directly developing individuals showed shorter development time, higher growth rate, and relatively lower body weight than diapausing individuals. There were no significant differences in weight losses between the direct and diapause development individuals. Our results highlight the importance of the expression of alternative developmental pathways in the evolution of life history.

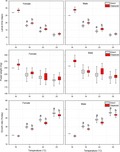

## 1 INTRODUCTION

It has been clearly recognized that diapause is an important mechanisms for synchronizing seasonal development and activity in subtropical and temperate zone insects. However, diapause has another important role that is often ignored—it permits the insects to breed dispersively at different periods, because their chances of survival are greatly enhanced (Danks, 1987; Masaki, 1980; Tauber, Tauber, & Masaki, 1986; Xue & Kallenborn, 1993). Portions of many insect populations are known to enter into diapause while the remaining insects continue to develop and reproduce. For example, 6 years of field observations of summer diapause in the zygaenid moth, *Pseudopidorus fasciata* have shown that only 20%–28% of individuals of the overwintering generation and 49%–60% of the first generation entered summer diapause as prepupae, while the rest continued to develop and produce the next generation (Xue & Kallenborn, 1998). In the fly, *Pegomyia bicolor*, 6 years of field observations indicated that 41%–70% of individuals that pupated during April 5–7 entered pupal diapause, while the rest continued to emerge and oviposit and produced the second generation (Xue, Zhu, & Shao, 2001). In the cabbage beetle, *Colaphellus bowringi*, there were always some individuals (17%–67%) hatched between August 5 and October 5 entering diapause as adults, while the rest mated and produced the next generation (Xue, Spieth, Li, & Hua, 2002). That part of the population entering dormancy (summer or winter diapause) and the rest the population whose development continues have been regarded to constitute a bet‐hedging or risk‐spreading reproductive strategy that avoids the situation in which individuals “put all their eggs into one basket” (Hopper, 1999; Masaki, 1980; Tauber et al., 1986; Waldbauer, 1978; Wise, 1980; Xue & Kallenborn, 1993) and subjects them all simultaneously to the possibility of meeting unfavorable environmental conditions. Individual insects that follow these two alternative pathways of diapause and direct development are typically described as presenting alternative developmental pathways (Aalberg Haugen & Gotthard, 2015; Fischer & Fiedler, 2000; Gotthard & Berger, 2010; Kivelä, Svensson, Tiwe, & Gotthard, 2015; Kivelä, Välimäki, & Gotthard, 2016; Kivelä, Välimäki, & Mäenpää, 2012). However, there have been a few studies showing that the expression of life‐history traits may differ between these pathways (Aalberg Haugen, Berger, & Gotthard, 2012; Aalberg Haugen & Gotthard, 2015; Chen, Xia, Xiao, Xiao, & Xue, 2014; Gotthard & Berger, 2010; Kivelä, Välimäki, & Gotthard, 2013). Understanding the expression of two alternative developmental pathways may be crucial for studying life history evolution and developing successful pest management programs (Nylin, 2001)**.**

The cabbage butterfly, *Pieris melete* Ménétriés is a serious pest of crucifers in the mountain areas of the Jiangxi Province, PR China and has a multivoltine life cycle with both summer and winter diapause in the pupal stage. The effects of temperature and photoperiod on diapause induction and termination have been evaluated in detail in this butterfly species under laboratory and field conditions (Xiao, Li, Wei, & Xue, 2008; Xiao, Wu, He, Chen, & Xue, 2012; Xiao, Wu, Wang, Zhu, & Xue, 2009; Xue, Kallenborn, & Wei, 1997). Under laboratory conditions, the photoperiodic response curves in *P. melete* showed an intermediate response type: that is, short day lengths (8–11 hr) induced winter diapause; the intermediate day lengths (12–13 hr) permitted some pupae to develop without diapause, and the long day lengths (14–16 hr) induced summer diapause (Xiao et al., 2009). These studies also revealed that high temperatures strongly weakened the diapause‐inducing effects of long day length and significantly reduced the incidence of summer diapause; whereas winter diapause can be induced under short day‐length at relatively high temperatures (Xiao et al., 2009; Xue et al., 1997). In the field, there are two distinct infestation peaks per year, one in the spring and a second in autumn. According to our field observations for 9 years (1988, 1989, 1994, 1995, 2003, 2004, 2005, 2006, and 2007), if the overwintered pupae eclosed into adults between mid‐March and early April (1988, 1989, 1994, 1995, 2003, 2005, and 2007), almost all their progenies would have entered summer diapause and produced one generation. However, if adults emerged between late February and late March, some progenies produced by the early emerged adults would have developed without diapauses (33.33% in 2004; 34.04% in 2006; Xiao et al., 2012), these progenies emerged as adults in late April and produced the second generation. In autumn, aestivating individuals emerge between the end of August and early November. Early‐emerging individuals can produce three generations in autumn under conditions of relatively high temperatures and intermediate day lengths. However, late‐emerging individuals produce only one generation because of the relatively low temperature and short day lengths occurring in late autumn. Thus, there are one to three generations in autumn (Xue, Zhu, & Wei, 1996). Furthermore, there are always some individuals entering winter diapause regardless of temperature, as indicated by the fact that 3.85% of individuals in 2003, 4.65% in 2004, and 6.78% of individuals in 2005 that hatched in August entered winter diapause even under high temperatures—from 26.4 to 31.2°C (Xiao et al., 2012).

Therefore, this insect species may serve as an excellent experimental model to test the differences in life history traits between the diapausing and directly developing individuals. In the present study, we tested the influence of temperature, day length, and seasonality on the expression of alternative developmental pathways in *P. melete* under laboratory and field conditions and their differences in larval and pupal development time, pupal weight and growth rate, and adult weight and weight loss, aiming to understand how temperature, day length, and seasonality affect the evolution of their life‐history traits.

## 2 MATERIALS AND METHODS

### 2.1 Experimental insects

The cabbage butterflies, *P. melete* used in the experiments originated from a wild population in the Tonggu County (28.5°N, 114.4°E; at an altitude of approximately 240 m above sea level), Jiangxi Province, PR China. Mature larvae prior to pupation were collected from crucifers in the vegetable gardens in mid‐November, 2015 and late April, 2016 and then were transferred to wooden insectaries (30 × 30 × 35 cm) for pupation and hibernation and estivation under natural conditions. Adults from the overwintering or aestivating pupae were released to an outdoor web‐screened insectary with cultivated flowering Chinese cabbage, *Brassica chinensis* for mating and oviposition in the spring or autumn, respectively. Eggs laid on leaves were collected in Petri dishes (height 2 cm; diameter 9.0 cm) lined with moistened filter paper every day and were used to conduct the experiments.

### 2.2 Experimental arrangement

#### 2.2.1 Laboratory experiments

After hatching, young larvae from the spring generation were reared in Petri dishes (height 2 cm; diameter 9.0 cm) containing moistened filter paper and fresh leaves of *B. chinensis* with four larvae in a Petri dish. The Petri dishes were randomly divided into four groups and were placed in four illuminated incubators (LRH‐250‐GS, Guangdong Medical Appliances Plant) with constant temperatures of 16, 19, 22, and 25°C. The photoperiod was identical in all treatments (24‐hr L/D cycle, L:D 12.5:11.5 hr). At least 30 Petri dishes were used for each temperature treatment. The Petri dishes were checked daily and supplied with new fresh leaves when needed. After pupation, pupae were placed individually in a transparent plastic box (3.5 cm in diameter and 6 cm in height) lined with filter paper and the box was covered with gauze. The pupae were monitored for eclosion to determine the developmental pathway for each individual. Based on the current experiment, if they did not emerge within 35 days at 16°C, 15 days at 19°C, 12 days at 22°C, and 9 days at 25°C, they were assumed to be in diapause. Diapause pupae were placed at 8°C for 30 days in continuous darkness and then transferred to L:D 15:9 hr and 18°C conditions to terminate the pupal diapause and observe the adult emergence. The number of females and males was recorded daily.

#### 2.2.2 Field experiments

Newly hatched larvae from the spring generation were transferred to *B. chinensis* plants grown in an outdoor web‐screened insectary. When the larvae matured they were placed individually in a transparent plastic box (3.5 cm in diameter and 6 cm in height) lined with filter paper and fresh leaves for pupation. After pupation, the pupae were transferred individually to a clean transparent plastic box for eclosion. Adults emerging from nondiapause pupae were released into another outdoor web‐screened insectary to mate and produce the second generation using the same protocol. Nondiapause pupae generally emerged within 8–13 days in the spring generations. Thus, each pupa that did not emerge within 15 days in spring generations was assumed to be in summer diapause. Similar to the actions during laboratory experiment, aestivating pupae were placed at 8°C (a low temperature that can accelerate the development of diapaus and shortened diapause duration) for 30 days in continuous darkness (Xiao, Wu, Chen, & Xue, 2013), and then transferred to LD 15:9 hr, 18°C conditions to terminate pupal diapause and observe the adult emergence. The number of females and males was recorded daily.

In the autumn generations, diapausing and nondiapausing pupae were obtained using the same method as that used for the spring generations. Nondiapause pupae generally emerged within 7–21 days in the autumn generations. Thus, each pupa that did not emerge within 25 days was assumed to be in diapause. The diapause pupae were treated under the same conditions as were the aestivating pupae to observe the adult emergence. The diapause pupae of the second autumn generation were maintained under natural conditions until adult eclosion the following spring.

The data of the mean daily temperature experienced by larvae for each generation were collected from the weather station of Jiangxi Agricultural University.

### 2.3 Measurement methods

For each diapausing and directly developing individual obtained from both laboratory and field conditions, we measured the larval and pupal development time from hatching to pupation and adult eclosion, pupal and adult weight, growth rate, and proportional weight loss at metamorphosis. We calculated the pupal weight on the 2nd day after pupation and adult weight after the release of the meconium by using an electronic balance (AUY120; Shimadzu). The individual growth rate of each larva used in the experiments was calculated according to the methods of Gotthard, Nylin, and Wiklund (1994): Growth rate = ln (pupal weight)/larval time × 100. This formula gives a relative growth rate representing the mean weight gain per day. Weight loss between pupation and adult eclosion was calculated using the following formula: proportion weight loss = 1 − (adult weight/pupal weight).

### 2.4 Statistical analyses

Statistical analyses were conducted using the SPSS 17.0 statistical software package (IBM, http://www.ibm.com). Life history traits were analyzed in relation to temperature, development pathway, and sex with the general linear model. The nonsignificant three‐way term (temperature‐by‐development pathway‐by‐sex) was dropped from the final model in the analysis. One‐way analysis of variance (ANOVA) was used to determine whether there were significant differences in life history traits in different development pathways at each temperature. One‐way analysis of variance and Duncan's test were used to compare the differences in life history traits between sexes in each development pathway and at each temperature. Throughout the text, all means are given with ±1 *SE*s.

## 3 RESULTS

### 3.1 Developmental pathway at constant temperatures

When larvae were reared under an intermediate day length of 12.5 hr, 98.2% of the pupae entered diapause at the low temperature of 16°C regardless of the favorable day length (Table 1). However, when larvae were reared at relatively high temperatures, some individuals underwent direct development. The percentage of directly developing pupae reached 44.0% at 19°C, 77.0% at 22°C, and 63.1% at 25°C. The intermediate day length of 12.5 hr combined with the intermediate temperature of 22°C promoted direct development most effectively.

**Table 1:** Incidence of developmental pathways taken by *Pieris melete* pupae at different constant temperatures

*T* (°C)	*N*	Developmental pathway		
		Direct	Diapause	Direct (%)
16	112	2	110	1.8
19	91	40	51	44.0
22	87	67	20	77.0
25	195	123	72	63.1

Note

The photoperiod (24‐hr L:D cycle, L:D 12.5:11.5) was identical in all treatments.

### 3.2 Comparisons of life‐history traits between diapausing and directly developing individuals at constant temperatures

**Figure 1:**
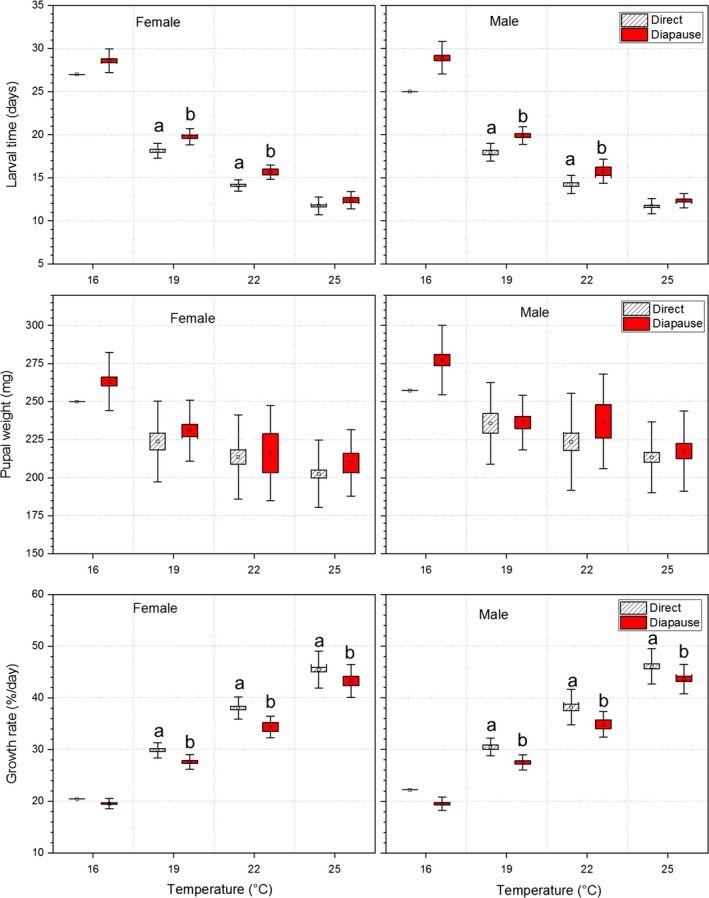
Comparisons of larval time, pupal weight, and growth rate between diapausing and directly developing individuals of *Pieris melete* at constant temperatures of 16, 19, 22, and 25°C. The symbols of ▫, □, and 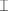 represent mean values, ± *SE*s and ± *SD*s, respectively. The values with different lowercase letters are significantly different between diapausing and directly developing individuals at a significant level of 0.05

Temperature, developmental pathway, and their interactions (temperature × developmental pathway) significantly affected larval development time (Table 2). Larval development time significantly decreased as rearing temperature increased, with the larval development time of directly developing individuals being shorter than that of diapausing individuals and with significant differences at 19 and 22°C (Figure 1, Table S1, *p* < .05).

**Table 2:** Results from a linear model analysis of fixed effects on larval time, pupal weight, growth rate, adult weight, and weight loss of *Pieris melete* at different constant temperatures in relation to temperature, development pathway (develop. path), and sex

Traits	Fixed effects	*df*	*F*	*p*
Larval time	Temp	3	2,222.199	**<.001**
	Develop. path.	1	72.072	<**.001**
	Sex	1	0.217	.641
	Temp × develop. path.	3	6.245	**.02**
	Temp × sex	3	0.295	.829
	Develop. path. × sex	1	0.106	.745
Pupal weight	Temp	3	59.346	**<0.001**
	Develop. path.	1	2.946	.087
	Sex	1	17.740	**<.001**
	Temp × develop. path.	3	0.109	.897
	Temp × sex	3	0.343	.795
	Develop. path. × sex	1	0.002	.960
Growth rate	Temp	3	1,028.836	**<.001**
	Develop. path.	1	60.015	**<.001**
	Sex	1	1.095	.296
	Temp × develop. path.	3	0.838	.433
	Temp × sex	3	0.077	.972
	Develop. path. × sex	1	0.064	.800
Adult weight	Temp	3	41.559	**<.001**
	Develop. path.	1	8.639	**.003**
	Sex	1	3.465	.063
	Temp × develop. path.	3	0.231	.794
	Temp × sex	3	0.165	.920
	Develop. path. × sex	1	0.116	.734
Weight loss	Temp	3	2.688	**.046**
	Develop. path.	1	3.357	.068
	Sex	1	3.837	.051
	Temp × develop. path.	2	1.576	.208
	Temp × sex	3	1.199	.310
	Develop. path. × sex	1	0.683	.409

Note

The significant effects are highlighted in bold.

Pupal weight was significantly affected by temperature and sex (Table 2). Pupal weight gradually decreased as rearing temperature increased from 16 to 25°C (Figure 1), which is consistent with the general pattern in ectothermic animals (Atkinson, 1994). Males were slightly larger than females at each temperature, but this difference was not significant (see Table S1, *p* > .05). Individuals that developed directly into adults attained relatively lower pupal weights than did individuals entering diapause (Figure 1), although significant differences were not found at each temperature (Table S1, *p* > .05).

The temperature and developmental pathway significantly affected larval growth rate (Table 2). The growth rate increased significantly as the rearing temperature increased. Individuals that developed directly into adults had significantly higher growth rates than individuals entering diapause (Figure 1, Table S1).

Adult weight was significantly affected by temperature and developmental pathway (Table 1).

**Figure 2:**
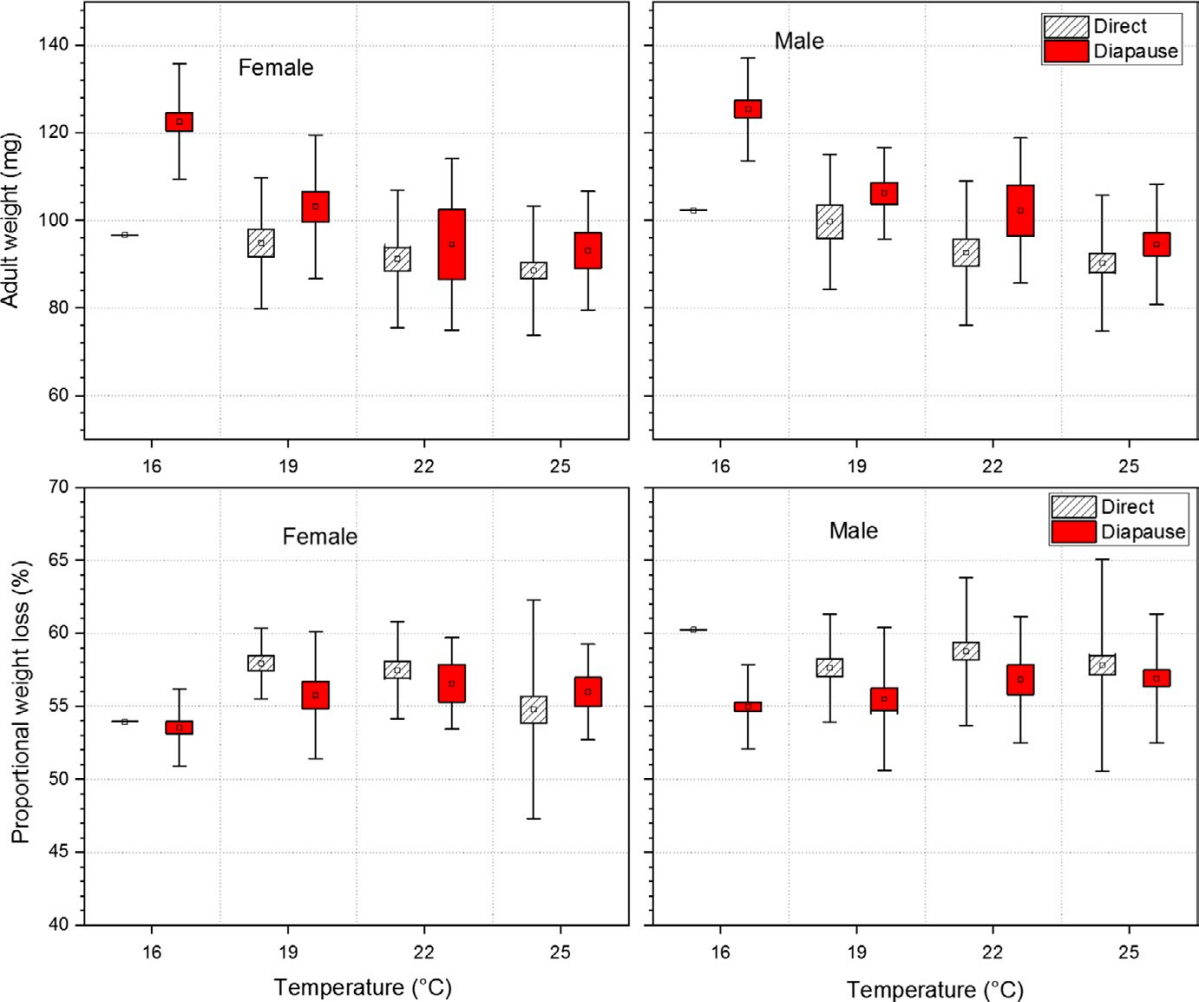
Comparisons of weight loss from pupae to adults between diapausing and directly developing individuals of *Pieris melete* at constant temperatures of 16, 19, 22, and 25°C. The symbols of ▫, □, and 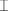 represent the mean value, ± *SE*s and ± *SD*s, respectively. The values with different lowercase letters are significantly different between diapausing and directly developing individuals at a significant level of 0.05

Adult weight gradually decreased as the rearing temperature increased (Figure 1). Although Table 1 shows a significant effect on adult weight induced by developmental pathway, there was no significant difference in adult weight between diapausing and directly developing individuals at any temperature, with diapausing individuals being slightly larger than directly developing individuals (Figure 2, Table S1). Male adults were slightly larger than female adults (Figure 2, Table S1).

Temperature significantly affected weight loss from pupa to adult (Table 1). The proportional weight losses of diapausing pupae were similar to those of directly developing individuals at all temperatures (55%–59%; see Table S1). There were no significant differences in weight loss between males and females at any temperature (Table S1).

### 3.3 Developmental pathways under field conditions

In the spring generations, almost all individuals entered summer diapause (97.1% in the first spring generation, 93.5% in the second spring generation), only a few individuals underwent direct development (Table 2). This is because they experienced relatively low mean daily temperatures and gradually prolonging day lengths. In the autumn generations, most individuals developed without diapause (90% in the first autumn generation, 78.5% in the second autumn generation) when they experienced gradually shortening day lengths (close to the intermediate day lengths) and relatively high mean daily temperatures (Table 3).

**Table 3:** Incidence of developmental pathways taken by *Pieris melete* pupae at different mean daily temperatures and altered day lengths in the outdoor screened insectary

Generation	The mean daily temperature (°C) and day length (hr) experienced by larvae	Date from hatching to pupation	*N*	Developmental pathway		
				Direct	Diapause	Direct (%)
Spring	16.8°C 12.6–13.5 hr	Mar 21–Apr 18	547	16	531	2.9
Spring	20.8°C 13.5–14.4 hr	Apr 22–May 23	307	20	287	6.5
Autumn	25.6°C 13.3–12.1 hr	Sept 3–Sept 23	50	45	5	90
Autumn	17.6°C 12.1–11.3 hr	Oct 16–Nov 15	121	95	26	78.5

### 3.4 Comparisons of life‐history traits between diapausing and directly developing individuals under field conditions

**Figure 3:**
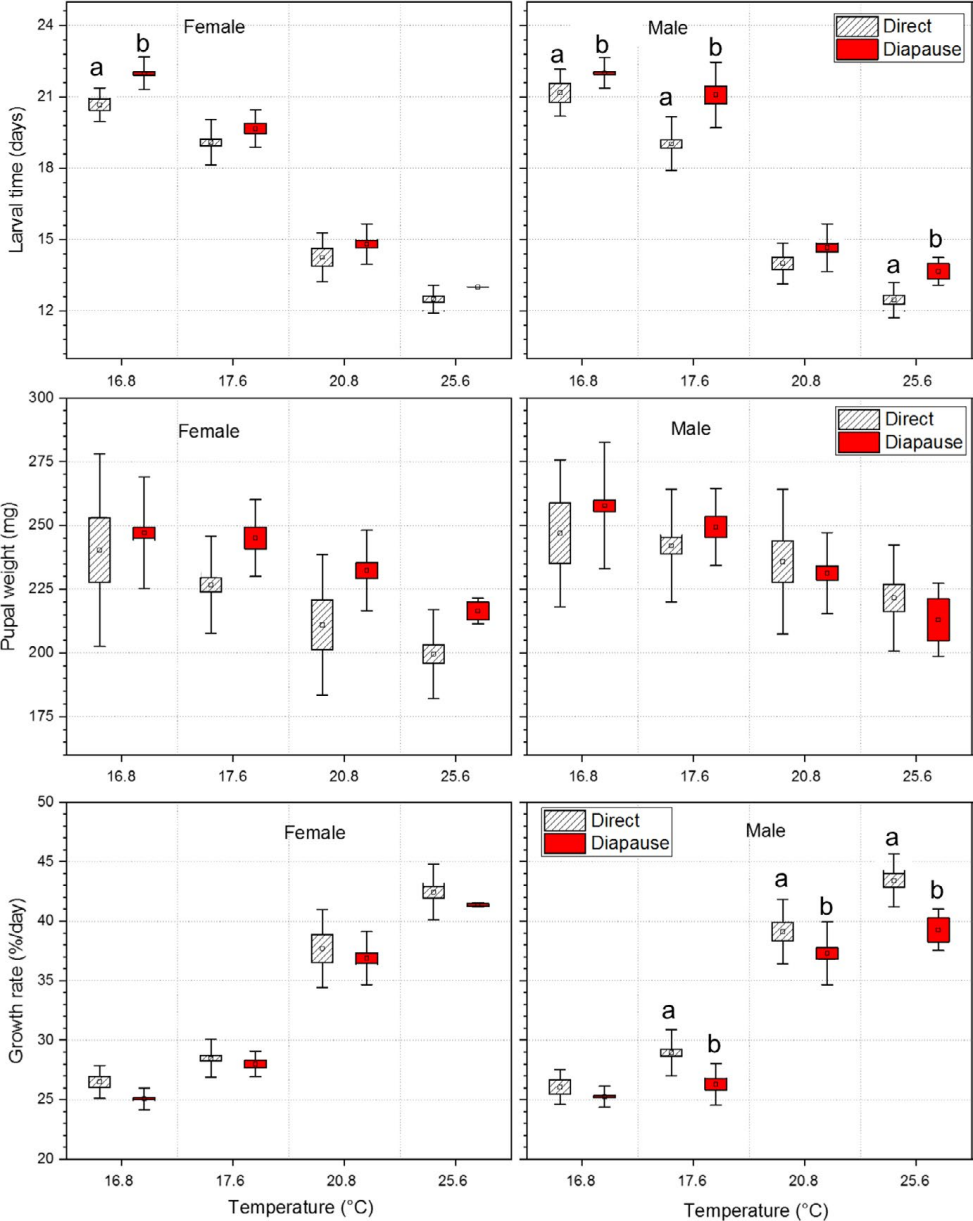
Comparisons of larval time, pupal weight, and growth rate between diapausing and directly developing individuals of *Pieris melete* at different mean daily temperatures in the field. The symbols of ▫, □, and 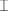 represent the mean value, ± *SE*s and ± *SD*s, respectively. The values with different lowercase letters are significantly different between diapausing and directly developing individuals at a significant level of 0.05. The mean daily temperatures of 16.8, 17.6, 20.8, and 25.6°C represent the first spring generation, the second autumn generation, the second spring generation, and the first autumn generation, respectively

Larval development time was significantly influenced by temperature and developmental pathway (Table 4). There were significant interactions (temperature × developmental pathway, temperature × sex and temperature × developmental pathway × sex). The larval development time significantly decreased with increasing the mean daily temperature. The larval development times were shorter in directly developing individuals than in diapausing individuals, with significant differences at 16.8°C for females and at 17.6 and 25.6°C for males (Figure 3, Table S2, *p* < .05).

**Table 4:** Results from a linear model analysis of fixed effects on larval time, pupal weight, growth rate, adult weight, and weight loss of *Pieris melete* under field conditions in relation to temperature, development pathway (develop. path), and sex

Traits	Fixed effects	*df*	*F*	*P*
Larval time	Temp	3	985.126	**<.001**
	Sex	1	3.695	.055
	Develop. path.	1	49.794	**<.001**
	Temp × sex	3	3.064	**.028**
	Temp × develop. path	3	2.105	.099
	Develop. path. × sex	1	2.712	.100
	Temp × develop. path. × sex	3	4.123	**.007**
Pupal weight	Temp	3	15.796	**<.001**
	Sex	1	7.603	**.006**
	Develop. path.	1	5.659	**.018**
	Temp × sex	3	0.053	.984
	Temp × develop. path	3	0.249	.862
	Develop. path. × sex	1	4.098	**.044**
Growth rate	Temp	3	827.492	**<.001**
	Sex	1	0.153	.696
	Develop. path.	1	37.210	**<.001**
	Temp × sex	3	2.542	.56
	Temp × develop. path	3	0.942	.420
	Develop. path. × sex	1	6.829	**.009**
Adult weight	Temp	3	15.255	**<.001**
	Sex	1	2.563	.197
	Develop. path.	1	5.414	**.02**
	Temp × sex	3	0.284	.837
	Temp × develop. path	3	0.012	.998
	Develop. path. × sex	1	2.243	.135
Weight loss	Temp	3	5.951	**.001**
	Sex	1	0.863	.353
	Develop. path.	1	1.719	.190
	Temp × sex	3	0.844	.470
	Temp × develop. path	3	0.576	.631
	Develop. path. × sex	1	0.000	.982

Note

The significant effects are highlighted in bold.

Pupal weight was significantly affected by temperature, sex, developmental pathway, and a significant interaction (developmental pathway × sex; Table 4). Pupal weight gradually decreased as the mean daily temperature increased, with diapausing individuals being slightly larger than directly developing individuals (Table S2). Male pupae were generally slightly larger than female pupae, with a significant difference at 20.8°C (Figure 3, Table S2, *p* < .05).

Growth rate was significantly affected by temperature and developmental pathway; developmental pathway and sex had a significant interaction (Table 4). The growth rate increased significantly as the mean daily temperature increased. Moreover, the growth rate was higher in directly developing individuals than diapausing individuals, showing significant differences for males at mean daily temperatures of 17.6, 20.8 and 35.6°C (Figure 3, Table S2, *p* < .05).

**Figure 4:**
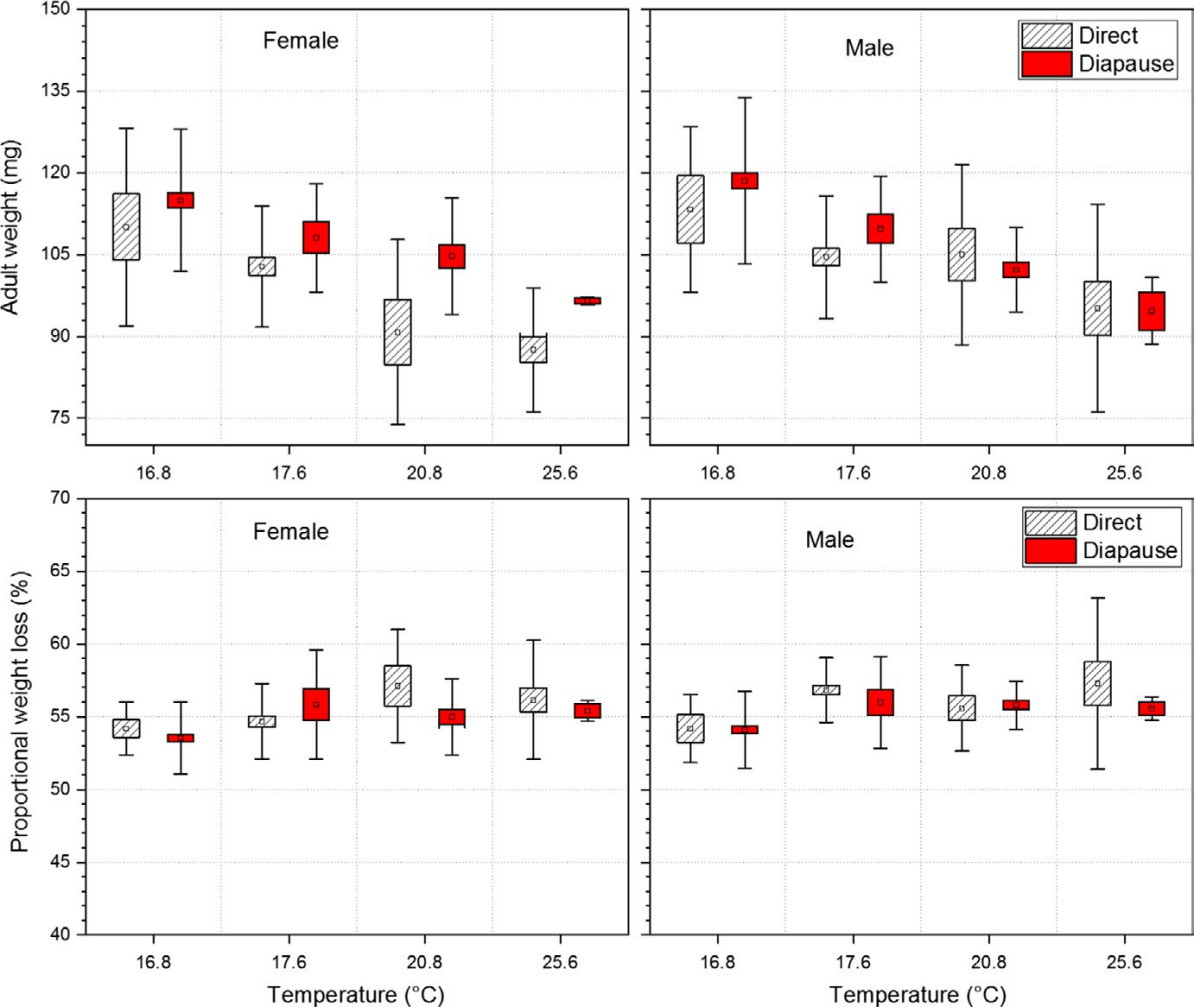
Comparisons of weight loss from pupae to adults between diapausing and directly developing individuals of *Pieris melete* at different mean daily temperatures in the field. The symbols of ▫, □, and 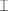 represent the mean values, ± *SE*s and ± *SD*s, respectively. The values with different lowercase letters are significantly different between diapausing and directly developing individuals at a significant level of 0.05. The mean daily temperatures of 16.8, 17.6, 20.8, and 25.6°C represent the first spring generation, the second autumn generation, the second spring generation, and the first autumn generation, respectively

Temperature and developmental pathway significantly affected adult weight (Table 4). Adult weight gradually decreased as the mean daily temperature increased (Figure 4), with diapausing individuals being slightly larger than directly developing individuals (Table S2). Male adults were larger than female adults, with a significant difference at 20.8°C (Figure 4, Table S2, *p* < .05).

Proportional weight loss from pupa to adult was significantly affected by temperature (Table 4). However, the proportional weight loss did not show significant differences between diapausing individuals and directly developing individuals at any temperature, although diapausing individuals experienced significantly longer durations of pupae than did directly developing individuals (67–97 vs. 7–13 days).

## 4 DISCUSSION

The experimental results in *P. melete* under both laboratory and field conditions showed similar patterns in that the expression of the developmental pathway is highly plastic, depending on temperature, day length, and their interaction (see Tables 1 and 3). Constant low temperatures of 16°C and the mean daily spring temperatures of 16.8 and 20.8°C combined with gradually increasing day lengths resulted in almost all individuals entering pupal diapause (98.2% and 97.1%). Higher constant temperatures (19, 22, and 25°C) and mean daily autumn temperatures (17.6 and 25.6°C) combined with gradually shortened autumn day lengths induced most individuals to develop without diapause (90% and 78.5%). In nature, such response patterns for diapause induction play an important role in regulating the life cycle of *P. melete*. The low‐temperature control of diapause not only ensures almost all larvae that grow in the spring enter summer diapause, thus avoiding reproduction during adverse summer conditions (e.g., drought and food shortage) but also ensures that that the species synchronize its development and reproduction with the growth seasons of the host plants (Xue et al., 1997). In the field, all cruciferous vegetables are harvested in May and are generally not cultivated until autumn. The relatively high autumn temperature induction of direct development enables the butterfly to maximize the use of the available food resources (approximately 3.5 months) for building up the population. The higher autumn temperature induction of diapause in some individuals reflects a bet‐hedging tactic that allows the butterfly to escape from various unpredictable physical or biotic factors, such as farming practices (insecticide applications, thinning of the seedlings and harvesting), interspecific competition (aphids and the beetle *Phaedon brassicae*), and autumn drought, thus avoiding the catastrophic elimination of an entire population.

Given the physiological responses that control the propensity to enter diapause, the expression of an alternative development pathway in *P. melete* may be dependent upon a threshold. Those individuals above a certain threshold value may enter diapause; those below it may avert diapause. Alternatively, alternative developmental pathways may entail that conditionally expressed genes, although carried by all individuals within a population, are expressed and exposed to selection only a fraction of these individuals at any given time (Van Dyken & Wade, 2010).

Individual insects that follow the two alternative pathways of diapause and direct development often display substantial phenotypic differences in life history traits. Directly developing individuals generally have shorter development times and higher growth rates than do diapausing individuals (Aalberg Haugen et al., 2012; Blanckenhorn & Fairbairn, 1995; Kivelä et al., 2012; Nylin, 1992; Pöykkö & Hyvärinen, 2012; Wiklund & Friberg, 2011; Wiklund, Nylin, & Forsberg, 1991). Compared to that of diapausing individuals, the body size of directly developing individuals varies with species and may be relatively larger (Kivelä et al., 2012; Wiklund et al., 1991), relatively smaller (Aalberg Haugen et al., 2012; Mousseau & Roff, 1989; Pöykkö & Hyvärinen, 2012; Teder, Esperk, Remmel, Sang, & Tammaru, 2010), or equal (Blanckenhorn, 1994; Blanckenhorn & Fairbairn, 1995; see also Välimäki, Kivelä, Mäenpää, & Tammaru, 2013). In the present study, we found that compared with the diapause pathway, the direct development pathway in *P. melete* is generally associated with shorter larval development times, higher larval growth rates and relatively lower pupal, and adult weights.

Under both laboratory and field conditions, pupal and adult weights for both diapausing and directly developing individuals of *P. melete* gradually decreased with increasing temperature, showing a typical thermal reaction norm for ectotherm body size, denoted as the temperature‐size rule (TSR; Atkinson, 1994). However, increasing evidence has shown that the reverse TSR in insects is also common. For example, reversals of the TSR have been found in four species of mayfly (Atkinson, 1995); four species of British grasshoppers (Willott & Hassall, 1998); the tropical butterfly, *Bicyclus anynana* (Fischer, Bot, & Brakefield, 2004); the small cabbage white, butterfly, *Pieris rapae* (Kingsolver, Massie, Ragland, & Smith, 2007); the Asian corn borer, *Ostrinia furnacalis* (He, Tang, Huang, Gao, & Xue, 2019; Xiao et al., 2016); and the rice stem borer, *Chilo suppressalis* (Fu, He, Zhou, Xiao, & Xue, 2016; Huang, Xiao, He, & Xue, 2018). As such, why do some insect species follow the TSR and some exhibit the reverse TSR? We speculate whether an insect species follows the TSR or not may be related to its diapause characteristic. Those species with summer diapause may exhibit the TSR, as indicated by the cabbage beetle, *C. bowringi* (Tang, He, Chen, Fu, & Xue, 2017) and this butterfly, *P. melete* because their reproductive periods occur in the spring and autumn and because these insects have experienced strong selection for body size under relatively low environmental temperatures during the process of evolution. Those species with winter diapause triggered by shortening day lengths combined with high autumn temperatures may exhibit the reverse TSR, as indicated by the Asian corn borer, *O. furnacalis* (He et al., 2019; Xiao et al., 2016), and the rice stem borer, *C. suppressalis* (Fu et al., 2016; Huang et al., 2018). These two species enter winter diapause in response to high autumn temperatures and experience strong selection for body size under warm conditions. Additional insect species with similar diapause characteristics will be investigated to confirm this speculation.

To date, few studies have tested the differences in weight loss between the diapausing and directly developing individuals. In the cotton bollworm, *Helicoverpa armigera*, proportional weight losses were slightly lower in diapausing individuals than in directly developing individuals at 20 and 22°C, but slightly higher at 25°C (Chen et al., 2014). In the present study, the proportional weight losses were similar between the diapausing and directly developing individuals under both laboratory and field conditions at the same temperature conditions, despite those diapausing individuals exhibiting much longer pupal durations than did the directly developing individuals (Tables S1 and S2). Such a case indicates that the process of diapause did not affect body weight change during metamorphosis. Furthermore, the weights of both female and male adults were still slightly higher in diapausing individuals than in the directly developing individuals. Thus, newly emerged female adults from diapause development should have relatively high fecundity than those from direct development because female fecundity is generally positively correlated with adult body weight when the number of eggs is assessed as lifetime fecundity under standard conditions or by dissection (Honek, 1993). Therefore, relatively large body sizes in diapausing individuals are generally considered to be adaptive because of their greater reserves (Hahn & Denlinger, 2007) and may ameliorate the negative cost of diapause.

## CONFLICT OF INTEREST

The authors declare that they have no conflict of interest.

## AUTHOR CONTRIBUTIONS

FS‐X conceived and designed the research. JJ‐T, HM‐H, and C‐Z conducted experiments and analyzed the data. L‐X and SH‐Wu wrote the manuscript. All authors read and approved the manuscript.

## Data Availability

Empirical data have been archived in DataDryad: https://doi.org/10.5061/dryad.s5n7t4d
