# Supplementary material for: Expression of alternative developmental pathways in the cabbage butterfly, Pieris melete and their differences in life history traits
Source: Ecol Evol. 2019 Oct 21;9(21):12311–21. doi: 10.1002/ece3.5731 (PMC6854102; doi:10.1002/ece3.5731)
Supplement: Supplementary file 1 [file ECE3-9-12311-s001.docx]

**Appendix**

**Table S1.** Life-history data (mean ± 1 SE) for female and male of *Peris melete* at different constant temperatures. Values followed by the same letter do not differ significantly among temperatures (One-way analysis of variance (ANOVA) and Duncan’s test, threshold for significance *P* < 0.05)

| T (°C) | Sex | Developmental pathway | N | Larval time | Pupal time | Pupal weight | Growth rate | Adult weight | Proportion weight loss |
| --- | --- | --- | --- | --- | --- | --- | --- | --- | --- |
| 16 | Female | Direct | 1 | 27 | 25 | 249.9 | 20.45 | 96.7 | 0.54 |
|  |  | Diapause | 40 | 28.6±0.2a | 96.8±2ab | 263.25±3.03ab | 19.54±0.16a | 122.57±2.09a | 0.54±0a |
|  | Male | Direct | 1 | 25 | 30 | 257.3 | 22.2 | 102.3 | 0.6 |
|  |  | Diapause | 39 | 28.9±0.3a | 89.4±1c | 277.34±3.66a | 19.53±0.2a | 125.37±1.98a | 0.55±0ab |
|  |  |  |  |  |  |  |  |  |  |
| 19 | Female | Direct | 23 | 18.1±0.2b | 12.3±0.3d | 223.83±5.55cde | 29.87±0.31b | 94.84±3.19bcd | 0.58±0.01bc |
|  |  | Diapause | 24 | 19.8±0.2c | 99.9±3.7a | 230.95±4.09cd | 27.6±0.3c | 103.1±3.5be | 0.56±0.01abc |
|  | Male | Direct | 17 | 17.9±0.3b | 12.6±0.4d | 235.74±6.55cd | 30.5±0.42b | 99.68±3.74bcde | 0.58±0.01bc |
|  |  | Diapause | 20 | 19.9±0.2c | 99.9±5a | 236.21±4.04cd | 27.51±0.32c | 106.16±2.47e | 0.55±0.01abc |
|  |  |  |  |  |  |  |  |  |  |
| 22 | Female | Direct | 35 | 14.1±0.1d | 9.3±0.1d | 213.62±4.69cde | 38.03±0.36d | 91.16±2.66cd | 0.57±0.01bc |
|  |  | Diapause | 6 | 15.7±0.3e | 93.3±3.3bc | 216.15±12.8cde | 34.34±0.87e | 94.5±7.99bcd | 0.57±0.01abc |
|  | Male | Direct | 31 | 14.2±0.2d | 9.4±0.2d | 223.62±5.73cde | 38.18±0.61d | 92.61±2.96bcd | 0.59±0.01c |
|  |  | Diapause | 8 | 15.8±0.5e | 82.4±4.3e | 236.98±11.01bd | 34.85±0.87e | 102.26±5.86bce | 0.57±0.01abc |
|  |  |  |  |  |  |  |  |  |  |
| 25 | Female | Direct | 71 | 11.8±0.1f | 7.7±0.1d | 202.44±2.63e | 45.46±0.42f | 88.53±1.77d | 0.55±0.01ab |
|  |  | Diapause | 12 | 12.4±0.3f | 70.8±2.4f | 209.69±6.29ce | 43.24±0.93g | 93.11±4.08bcd | 0.56±0.01abc |
|  | Male | Direct | 52 | 11.7±0.1f | 7.5±0.1d | 213.34±3.22cde | 46.06±0.48f | 90.23±2.14d | 0.58±0.01bc |
|  |  | Diapause | 28 | 12.4±0.2f | 73.6±3.5f | 217.44±4.95cde | 43.66±0.54g | 94.51±2.64bcd | 0.57±0.01abc |

**Table S2.** Life-history data (mean ± 1 SE) for female and male of *Peris melete* at different mean daily temperatures in the field. Values followed by the same letter do not differ significantly among temperatures (One-way analysis of variance (ANOVA) and Duncan’s test, threshold for significance *P* < 0.05)

| T (°C) | Sex | Developmental pathway | N | Larval time | Pupal time | Pupal weight | Growth rate | Adult weight | Proportion weight loss |
| --- | --- | --- | --- | --- | --- | --- | --- | --- | --- |
| 16.8 | Female | Direct | 9 | 20.7±0.2a | 10±0.47a | 240.29±12.58abc | 26.5±0.5ab | 110.08±6.04abc | 0.54±0.01abc |
|  |  | Diapause | 96 | 22±0.1b | 92.17±3.21b | 247.13±2.22ab | 25.1±0.1a | 114.93±1.33ab | 0.54±0a |
|  | Male | Direct | 6 | 21.2±0.4a | 9.67±0.42a | 246.95±11.77ab | 26.1±0.6a | 113.28±6.19ab | 0.54±0.01abc |
|  |  | Diapause | 117 | 22±0.1b | 90.02±3.2bc | 257.76±2.29a | 25.2±0.1a | 118.56±1.41a | 0.54±0ab |
|  |  |  |  |  |  |  |  |  |  |
| 17.6 | Female | Direct | 46 | 19.1±0.1g | 13.24±0.08a | 226.68±2.81bcdf | 28.5±0.2g | 102.81±1.64bcdf | 0.55±0abc |
|  |  | Diapause | 12 | 19.7±0.2g | 93.67±1.86b | 245.02±4.34ab | 28±0.3bg | 108.11±2.87abcf | 0.56±0.01abc |
|  | Male | Direct | 49 | 19±0.2g | 13.16±0.14a | 242.04±3.16abc | 28.9±0.3g | 104.51±1.6abcdf | 0.57±0bc |
|  |  | Diapause | 13 | 21.1±0.4a | 94±1.22b | 249.4±4.2ab | 26.3±0.5a | 109.7±2.69abc | 0.56±0.01abc |
|  |  |  |  |  |  |  |  |  |  |
| 20.8 | Female | Direct | 8 | 14.3±0.4cd | 9.75±0.31a | 211.04±9.78de | 37.7±1.2cd | 90.81±5.99de | 0.57±0.01bc |
|  |  | Diapause | 26 | 14.8±0.2c | 96.85±4.63b | 232.33±3.09bcdf | 36.9±0.4c | 104.7±2.1abcdf | 0.55±0.01abc |
|  | Male | Direct | 12 | 14±0.2cd | 8.75±0.22a | 235.78±8.18abcf | 39.1±0.8d | 104.99±4.76abcf | 0.56±0.01abc |
|  |  | Diapause | 32 | 14.7±0.2c | 106.81±5b | 231.31±2.81bcdf | 37.3±0.5c | 102.21±1.36bcdf | 0.56±0abc |
|  |  |  |  |  |  |  |  |  |  |
| 25.6 | Female | Direct | 24 | 12.5±0.1e | 7.25±0.12a | 199.58±3.58e | 42.4±0.5ef | 87.56±2.32e | 0.56±0.01abc |
|  |  | Diapause | 2 | 13±0ef | 68.5±0.5c | 216.45±3.55def | 41.4±0.1e | 96.5±0.5cdef | 0.55±0.01abc |
|  | Male | Direct | 15 | 12.5±0.2e | 7.53±0.13a | 221.6±5.37cdef | 43.4±0.6f | 95.2±4.92def | 0.57±0.02c |
|  |  | Diapause | 3 | 13.7±0.3df | 67.33±1.2c | 213±8.29def | 39.3±1d | 94.67±3.53def | 0.56±0abc |
